# How Gastrin-Releasing Peptide Opens the Spinal Gate for Itch

**DOI:** 10.1016/j.neuron.2019.04.022

**Published:** 2019-07-03

**Authors:** Martina Pagani, Gioele W. Albisetti, Nandhini Sivakumar, Hendrik Wildner, Mirko Santello, Helge C. Johannssen, Hanns Ulrich Zeilhofer

**Affiliations:** 1 Institute of Pharmacology and Toxicology, University of Zurich, Winterthurerstrasse 190, 8057 Zurich, Switzerland; 2 Neuroscience Center Zurich, Winterthurerstrasse 190, 8057 Zurich, Switzerland; 3 Drug Discovery Network Zurich, Winterthurerstrasse 190, 8057 Zurich, Switzerland; 4 Institute of Pharmaceutical Sciences, Swiss Federal Institute of Technology (ETH) Zurich, Vladimir-Prelog-Weg 1-5/10, 8090 Zurich, Switzerland

**Keywords:** pruritus, optogenetics, GRP receptor, dorsal horn, interneuron, synaptic transmission, co-transmission, neuropeptide, volume transmission, sensory processing

## Abstract

Spinal transmission of pruritoceptive (itch) signals requires transneuronal signaling by gastrin-releasing peptide (GRP) produced by a subpopulation of dorsal horn excitatory interneurons. These neurons also express the glutamatergic marker vGluT2, raising the question of why glutamate alone is insufficient for spinal itch relay. Using optogenetics together with slice electrophysiology and mouse behavior, we demonstrate that baseline synaptic coupling between GRP and GRP receptor (GRPR) neurons is too weak for suprathreshold excitation. Only when we mimicked the endogenous firing of GRP neurons and stimulated them repetitively to fire bursts of action potentials did GRPR neurons depolarize progressively and become excitable by GRP neurons. GRPR but not glutamate receptor antagonism prevented this action. Provoking itch-like behavior by optogenetic activation of spinal GRP neurons required similar stimulation paradigms. These results establish a spinal gating mechanism for itch that requires sustained repetitive activity of presynaptic GRP neurons and postsynaptic GRP signaling to drive GRPR neuron output.

## Highlights

• Spinal itch relay requires effective communication from GRP to GRP receptor neurons
• Single action potentials in GRP neurons fail to release sufficient GRP
• Only burst firing releases enough GRP to prime GRP receptor neurons for activation
• GRP acts as a volume transmitter probably explaining why itch is hard to localize

## Graphical Abstract

**Figure:**
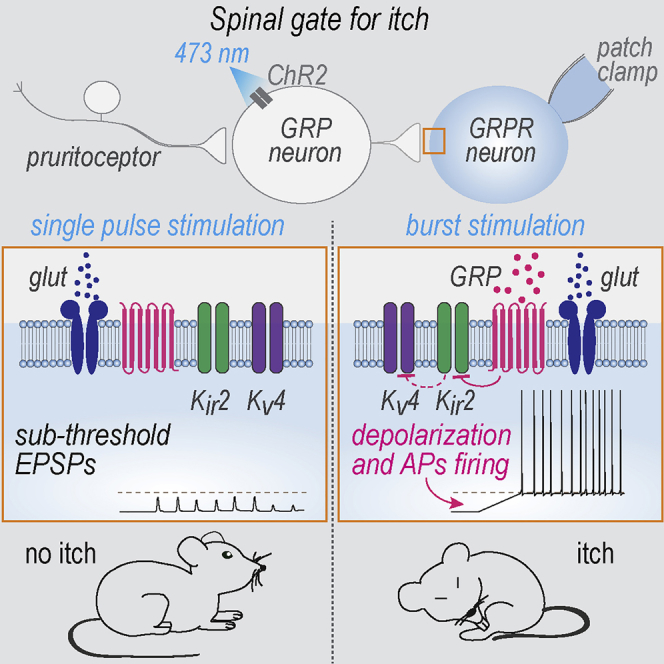

## Teaser

Pagani et al. investigated the temporal and spatial signature of GRP signaling, a key element of spinal itch relay. They find that GRP signals build up slowly and rather diffusely during stimulation, possibly explaining why itch is difficult to localize.

## Introduction

The senses of pain and itch have evolved to protect organisms from potentially harmful agents and stimuli (Yosipovitch et al., 2007). While the exposure to acute painful stimuli typically evokes an almost immediate withdrawal reflex and a fast onset pain sensation, pruritogens elicit a more prolonged “waxing and waning” sensation (Forster and Handwerker, 2014) and a less precisely timed scratching response aimed at the removal of the irritant.

Reponses to both types of stimuli are initiated by the activation of different types of specialized sensory nerve fibers, called nociceptors and pruritoceptors, which convey sensory information to the spinal or medullary dorsal horn. Despite the presence of neuropeptides such as substance P and calcitonin-gene-related peptide (CGRP) in peripheral and spinal pain pathways, plenty of evidence indicates that the responses to acute painful stimulation depend primarily on fast glutamatergic excitation of spinal cord neurons (Ault and Hildebrand, 1993, Lagerström et al., 2011, Liu et al., 2010, Olivar and Laird, 1999). By contrast, spinal transmission of itch signals is critically dependent on neuropeptide signaling. Several neuropeptides are expressed by peripheral pruritoceptors, including B-type natriuretic peptide (Huang et al., 2018, Mishra and Hoon, 2013) and neuromedin B (Goswami et al., 2014, Wan et al., 2017). These are likely released in the spinal cord, but it is at present unclear whether they are required for efficient itch relay to central (spinal cord) neurons. However, it is well established that the downstream relay of pruritoceptive signals from second-order to third-order dorsal horn interneurons is highly dependent on neuropeptide signaling by gastrin-releasing peptide (GRP), a 27-amino acid neuropeptide of the bombesin family (Majumdar and Weber, 2011). Mice lacking the GRP receptor (GRPR) exhibit strongly reduced responses to histamine-dependent and histamine-independent pruritogens (Sun and Chen, 2007), and local spinal ablation of the neurons that express the GRPR (GRPR neurons) almost completely protects mice from pruritus evoked by a broad variety of pruritogens (Sun et al., 2009). While a critical contribution of GRP-expressing neurons to spinal itch relay is undoubted (Albisetti et al., 2019, Sun et al., 2017), significant controversy exists about the identity and localization of the neurons that release GRP onto GRPR neurons (Gutierrez-Mecinas et al., 2014, Liu et al., 2014, Mishra and Hoon, 2013, Solorzano et al., 2015, Sun and Chen, 2007). The currently prevailing concept of spinal pruritoceptive signal transmission suggests that second-order dorsal horn interneurons that are activated by peripheral pruritoceptive neurons release GRP and in turn excite third-order (GRPR) interneurons that finally transmit pruritoceptive signals to spinoparabrachial (fourth-order) projection neurons (Goswami et al., 2014, Huang et al., 2018, Mu et al., 2017).

The dorsal horn neurons that express GRP comprise a population of excitatory interneurons that are located together with GRPR neurons in lamina II of the spinal dorsal horn (Albisetti et al., 2019, Gutierrez-Mecinas et al., 2016). These GRP neurons express, in addition to GRP, the vesicular glutamate transporter vGluT2 that confers a glutamatergic phenotype to these neurons (Gutierrez-Mecinas et al., 2014, Sun et al., 2017). The presence of vGluT2 hence raises the question why deletion of the GRPR gene or blockade of GRPR signaling has such a strong effect on itch behavior. In the present study, we have used optogenetics in slices and in freely behaving mice to demonstrate that GRP and GRPR neurons are indeed coupled via fast glutamatergic synapses. Yet, for efficient suprathreshold activation of GRPR neurons sufficient GRP release was found to be indispensable. Such GRP release was only achieved during repetitive burst-like activation of GRP neurons. These findings explain why itch strongly depends on neuropeptide signaling. They may also offer an explanation why itch and pain occur with strikingly different time courses.

## Results

### Neurochemical and Biophysical Analysis of GRP and GRPR Neurons

**Figure 1:**
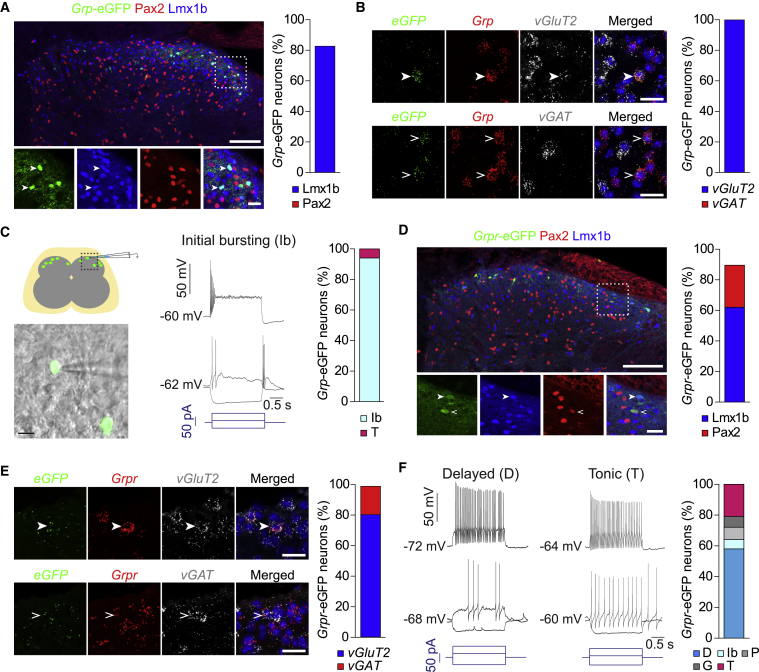
Physiological and Neurochemical Characteristics of Dorsal Horn GRP and GRPR Neurons (A) Transverse section of the lumbar dorsal horn of a *Grp*::eGFP mouse immunostained against eGFP, Pax2, and Lmx1b. Arrowheads indicate Lmx1b-positive *Grp*-eGFP neurons. Scale bars, 100 μm (overview) and 20 μm (high-magnification images). Bar chart: percentage of *Grp*-eGFP neurons positive for Lmx1b or Pax2 (n = 9 sections from 3 mice). (B) *In situ* hybridization for *vGluT2* and *vGAT* mRNA on dorsal horn sections of *Grp*::eGFP mice (merged image, DAPI in blue). Filled arrowheads, co-expression of *vGluT2* with *Grp*-*eGFP* and *Grp* mRNA; open arrowheads, lack of *vGAT* expression in *Grp*-eGFP neurons. Scale bar, 20 μm. Bar chart: percentage of *Grp*-*eGFP* neurons positive for *vGluT2* (43 out of 43 cells from 2 mice) and *vGAT* mRNA (0 out of 49 cells from 2 mice). (C) Left: experimental setup used for targeted recordings from dorsal horn neurons identified by eGFP fluorescence, and superimposition of a bright field and an epifluorescence image showing two *Grp*-eGFP neurons and a recording pipette. Scale bar, 10 μm. Right: voltage traces recorded from a *Grp*-eGFP neuron during somatic current injection. Bar chart: incidence of different firing patterns (n = 31 neurons from 12 animals). Ib, initial burst; T, tonic; D, delayed; G, gap; P, phasic firing. (D) Same as (A) but GRPR neurons (n = 15 sections from 5 mice). Open and filled arrowheads indicate Lmx1b-positive and Pax2-positive *Grpr*-eGFP neurons, respectively. (E) Same as (B) but *Grpr*-*eGFP* neurons. Bar chart: incidence of *Grpr-eGFP* neurons positive for *vGluT2* (29 out of 36 cells from 4 mice) and *vGAT* mRNA (5 out of 27 cells from 4 mice). Filled and open arrowheads indicate *Grpr*-*eGFP* neurons positive for *vGluT2* or *vGAT*, respectively. (F) Same as (C) but GRPR neurons (n = 91 cells from 61 mice).

To investigate synaptic communication between GRP and GRPR neurons, we used *Grp*::eGFP, *Grp*::cre, and *Grpr*::eGFP bacterial artificial chromosome (BAC) transgenic mice (all from Gensat, http://www.gensat.org/index.html). Eutrophic expression of eGFP in *Grp*::eGFP and of cre in *Grp*::cre mice has been reported previously (Gutierrez-Mecinas et al., 2014, Solorzano et al., 2015, Sun et al., 2017). Since *Grpr*::eGFP mice have not been systematically analyzed before, we used *in situ* hybridization on spinal cord sections to demonstrate that *eGFP* mRNA was restricted to *Grpr* mRNA-positive neurons (Figure S1). We then investigated biophysical and neurochemical characteristics of GRP and GRPR neurons in *Grp*::eGFP and *Grpr*::eGFP BAC transgenic mice (Figure 1). eGFP-expressing GRP and GRPR neurons were both concentrated in lamina II of the spinal dorsal horn. In accordance with a previous report (Gutierrez-Mecinas et al., 2014), staining with antibodies against Lmx1b and Pax2, respective markers of excitatory and inhibitory neurons, revealed that 83% ± 4% of *Grp*-eGFP neurons co-expressed Lmx1b, whereas no overlap with Pax2 was detected (Figure 1A). *In situ* hybridization experiments with probes directed against *vGluT2* and *vGAT*, marker genes of excitatory and inhibitory neurons, respectively, provided further support of a virtually exclusive excitatory phenotype of GRP neurons (Figure 1B). Consistent with other recently published data (Dickie et al., 2019), most of the *Grp*-eGFP neurons exhibited initial burst (Ib) firing, sometimes also referred to as transient firing (Yasaka et al., 2010), upon depolarizing current injection (Figure 1C). In dorsal horn neurons, this firing pattern is about equally abundant in excitatory and inhibitory neurons (Yasaka et al., 2010). Only few neurons (6%) responded with tonic firing that is much more frequent in the inhibitory dorsal neurons (Punnakkal et al., 2014). Unlike GRP-neurons, GRPR neurons were rather heterogeneous. Only 62% ± 4% of them co-expressed Lmx1b, while 27% ± 3% expressed the inhibitory marker Pax2 (Figure 1D). *In situ* hybridization analyses of *vGluT2* and *vGAT* revealed that 81% of *Grpr*-eGFP neurons were excitatory and 19% inhibitory neurons (Figure 1E). This heterogeneity was reflected by the firing patterns (Figure 1F). The majority of GRPR neurons (58%) responded with delayed action potential firing, but 21% showed tonic firing. Initial burst firing, phasic firing, and gap firing were also observed and each occurred in 6%–8% of the recorded neurons. These results suggest that GRPR neurons can be divided into a more abundant delayed firing and a less abundant tonic firing subtype. To correlate these firing patterns with either an excitatory or an inhibitory phenotype, we filled delayed and tonic firing *Grpr*-eGFP neurons during whole-cell recording with biocytin (1.5 mg/mL) and stained them post hoc for expression of Tlx3 (another marker of excitatory dorsal horn neurons) and Pax2 (Figure S2). All ten delayed firing neurons were Tlx3-positive, and 5 out of 6 tonic firing neurons expressed Pax2 (χ^2^ test; p = 0.0004). In the subsequent text, we refer to the delayed and tonic firing GRPR neurons as GRPR_excit_ and GRPR_inhib_ neurons.

GRP and GRPR neurons differed not only in their neurotransmitter phenotypes but also in their biophysical properties (Table 1). GRP neurons had more depolarized resting membrane potentials (RMPs) (−65.9 ± 1.2 mV versus −72.7 ± 0.6 mV), more depolarized action potential thresholds (−38.4 ± 0.5 mV versus −42.3 ± 0.42 mV), higher input resistances (1682 ± 104 MΩ versus 1050 ± 39.7 MΩ), and smaller rheobases (12.6 ± 1.2 pA versus 22.9 ± 1.6 pA) than GRPR neurons (for statistical comparisons see Table 1). These differences indicate a higher excitability of GRP neurons compared to GRPR neurons. In general, the differences to GRP neurons were more pronounced in the delayed firing GRPR_excit_ neurons than in the tonic firing GRPR_inhib_ neurons. Most strikingly, we observed a 3.5-fold larger rheobase in delayed firing GRPR_excit_ versus tonic firing GRPR_inhib_ neurons, suggesting that these neurons should be much less excitable than the tonic firing GRPR_inhib_ neurons. In addition, GRP neurons also had broader action potentials compared to GRPR delayed and tonic firing neurons.

**Table 1:** Passive and Active Biophysical Properties of *Grp*-eGFP and *Grpr*-eGFP Neurons

	RMP (mV)	C_m_ (pF)	R_input_ (MΩ)	Rheobase (pA)	Action Potential			
					Threshold (mV)	Amplitude (mV)	Width (ms)^a^	AHP (mV)
GRP (n = 31)	−65.9 ± 1.2	37.0 ± 2.3	1682 ± 104	12.6 ± 1.2	−38.4 ± 0.5	69.0 ± 1.7	3.71 ± 0.14	−29.6 ± 0.8
Versus GRPR delayed	^∗∗∗^	—	^∗∗∗^	^∗∗∗^	^∗∗^	—	^∗∗∗^	—
Versus GRPR tonic	—	—	^∗^	—	^∗∗∗^	—	^∗∗∗^	—
Versus GRPR phasic	^∗∗∗^	—	^∗∗∗^	^∗^	^∗∗∗^	—	^∗∗∗^	—
Versus GRPR gap	—	—	—	—	—	—	^∗∗∗^	^∗^
Versus GRPR initial bursting	—	—	—	—	—	—	^∗∗^	—
GRPR delayed (n = 53)	−73.3 ± 0.7	40.9 ± 2.0	1064 ± 49	28.1 ± 2.1	−41.5 ± 0.4	67.0 ± 2.8	2.18 ± 0.05	−29.1 ± 0.7
Versus GRPR tonic	—	—	^∗∗∗^	^∗∗∗^	—	—	—	—
Versus GRPR phasic	—	—	^∗∗∗^	—	^∗∗∗^	—	—	—
Versus GRPR gap	—	—	^∗∗∗^	—	—	—	—	^∗^
Versus GRPR initial bursting	—	—	^∗∗∗^	—	—	—	—	—
GRPR tonic (n = 19)	−70.2 ± 0.9	44.8 ± 3.7	1197 ± 92	8.21 ± 1.59	−43.8 ± 1.2	78.4 ± 1.5	2.22 ± 0.10	−28.5 ± 1.7
Versus GRPR phasic	—	—	^∗∗^	^∗∗^	—	—	—	—
Versus GRPR gap	—	—	—	—	—	—	—	^∗^
Versus GRPR initial bursting	—	—	—	—	—	—	—	—
GRPR phasic (n = 7)	−77.5 ± 2.8	35.6 ± 6.5	650 ± 160	28.3 ± 5.1	−47.4 ± 1.3	75.2 ± 3.2	1.83 ± 0.15	−23.8 ± 3.0
Versus GRPR gap	—	—	—	—	—	—	—	^∗∗∗^
Versus GRPR initial bursting	—	—	—	—	^∗∗^	—	^∗^	—
GRPR gap (n = 6)	−71.0 ± 2.2	48.5 ± 8.7	1001 ± 119	20.0 ± 3.6	−41.8 ± 1.0	80.6 ± 1.5	1.98 ± 0.15	−37.1 ± 2.0
Versus GRPR initial bursting	—	—	—	—	—	—	—	—
GRPR initial bursting (n = 6)	−70.4 ± 1.8	43.4 ± 4.2	990 ± 127	20.17 ± 3.6	−39.7 ± 1.5	62.0 ± 7.5	2.85 ± 0.26	−27.9 ± 3.3

RMP, resting membrane potential, C_m_, membrane capacitance, R_input_, input resistance, AHP, afterhyperpolarization. Values are means ± SEM. One-way ANOVA followed by a Bonferroni post hoc test. ^∗^p < 0.05, ^∗∗^p < 0.01, ^∗∗∗^p < 0.001. F(5,116) = 9.24 (RMP); 1.23 (Cm); 162 (R_i_); 11.85 (rheobase); 10.6 (AP threshold); 2.622 (action potential amplitude); 40.08 (action potential width); 3.90 (afterhyperpolarization).

a Determined at the action potential base.

### Synaptic Communication between GRP and GRPR Neurons

**Figure 2:**
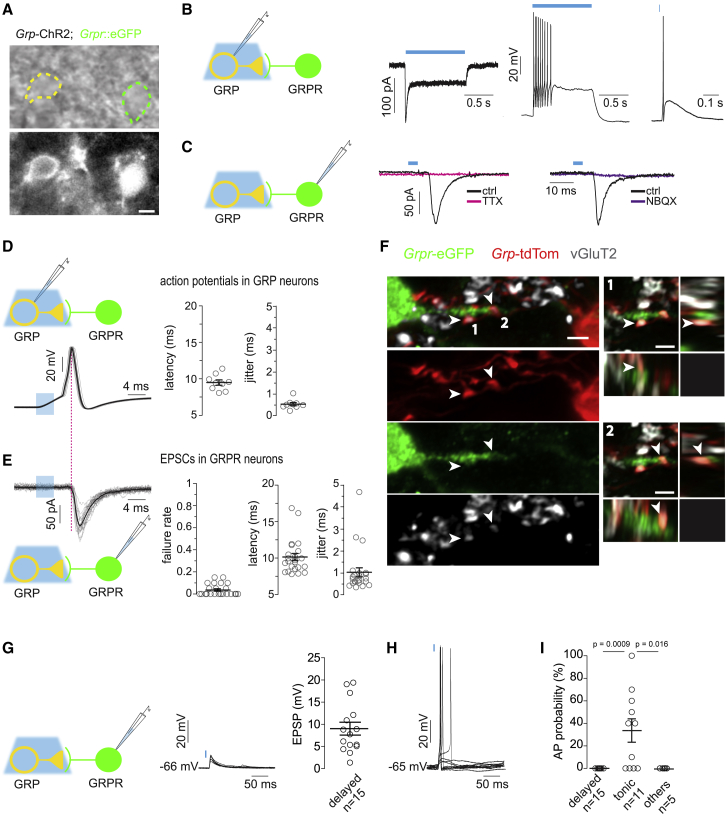
Synaptic Transmission between GRP and GRPR Neurons (A) Bright field (top) and epifluorescence (bottom) images of ChR2-eYFP- and *Grpr*-eGFP-positive neurons in a transverse lumbar spinal cord slice of a *Grp*-ChR2;*Grpr*::eGFP mouse. Scale bar, 5 μm. (B) Left: experimental setup. Middle: light-evoked photocurrent (473 nm, 1 s) recorded from an initial burst firing *Grp*-ChR2 neuron (yellow). Right: single blue light pulse-evoked (473 nm, 4 ms) action potential. (C) Left: experimental setup. Middle: light-evoked EPSCs before (black) and after 1 μM TTX application (magenta) recorded from a *Grpr*-eGFP neuron. Right: same as left but before (black) and after 20 μM NBQX (purple). Traces are averages of five consecutive responses. (D) Left: superposition of twenty consecutive light-evoked action potential traces (gray) recorded from a *Grp*-ChR2 neuron, average response (black). Light stimulation: 473 nm, 4 ms, 0.1 Hz. Right: latency and jitter of light-evoked action potentials recorded from *Grp*-ChR2 neurons (n = 9, from 7 animals). (E) Left: twenty consecutive EPSCs traces recorded from a *Grpr*-eGFP neuron. Right: failure rate, synaptic latency, and jitter of light-evoked EPSCs recorded from GRPR neurons (n = 23 cells from 14 animals). (F) Left: sagittal lumbar spinal cord section prepared from a *Grp*-tdTom;*Grpr*::eGFP mouse immunostained for tdTomato, eGFP, and vGluT2. Arrowheads indicate two examples of vGluT2-positive *Grp*-tdTom synaptic terminals contacting a *Grpr*-eGFP neuron dendrite. Right: single focal planes with the corresponding XZ (bottom) and YZ (right) orthogonal views from the same confocal z stack. All scale bars are 2 μm. (G) Light-evoked EPSPs recorded from delayed firing GRPR_excit_ neurons. Left: experimental setup. Middle: superposition of 10 consecutive light-evoked EPSPs. Right: EPSP amplitudes of 15 cells. (H) Same as (G) but tonic firing GRPR_inhib_ neurons. (I) Categorical scatterplot showing probabilities of light-evoked action potentials in different GRPR neuron subclasses (n = 30 cells from 28 animals). One-way ANOVA followed by Bonferroni post hoc test. F(2,28) = 9.48. p = 0.0007. All error bars indicate SEM. Circles denote values of individual cells.

We next studied synaptic communication between GRP and GRPR neurons. To this end, we made use of *Grp*::cre;Ai32;*Grpr*::eGFP triple transgenic mice (short *Grp*-ChR2;*Grpr*::eGFP mice). These mice express a channelrhodopsin2-eYFP fusion protein (ChR2-eYFP) in the cell membrane of GRP neurons and eGFP in the cytoplasm of GRPR neurons, allowing targeted recordings from GRPR neurons combined with optogenetic excitation of GRP neurons (Figures 2A and 2C). We first verified the presence of a blue light-evoked photocurrent in *Grp*-ChR2 neurons. After switching to current-clamp mode, 1 s blue light exposure induced an initial burst-firing pattern similar to the one we had observed previously in response to depolarizing current injections. Shorter (4 ms) light exposure induced a single action potential (Figure 2B). We then analyzed synaptic transmission between *Grp*-ChR2 and GRPR neurons (Figure 2C). We recorded evoked excitatory postsynaptic currents (EPSCs) in GRPR neurons upon wide-field illumination of the slice with short (4 ms) blue light pulses. On average, light-evoked EPSCs had amplitudes of −62.7 ± 6.8 pA (n = 23). Superfusion of the slice with tetrodotoxin (TTX, 1 μM, n = 5) or NBQX (20 μM, n = 7) caused a nearly complete block of EPSCs indicating that the recorded EPSCs depended on presynaptic action potentials and on postsynaptic activation of ionotropic glutamate receptors. We next compared latencies and jitter of light-evoked action potentials in GRP with those of light-evoked EPSCs in GRPR neurons (Figures 2D and 2E). Action potentials occurred with latencies of 9.5 ± 0.4 ms (n = 9) and with a low jitter (variability in latency: 0.51 ± 0.07 ms; n = 9). Light-evoked EPSCs in the postsynaptic GRPR neurons occurred with only slightly longer latencies (10.2 ± 0.5 ms; n = 23) than the action potentials in GRP neurons and also with a low jitter (1.0 ± 0.2 ms). Together with the very low failure rate, these findings suggest monosynaptic connections between the two neuron types. The monosynaptic nature of these connections was also supported by confocal microscopy performed in spinal cord sections of *Grp*::cre;Ai14;*Grpr*::eGFP (short *Grp*-tdTom;*Grpr*::eGFP) mice. These analyses revealed vGluT2-positive GRP-tdTom terminals in close opposition of *Grpr*-eGFP dendrites (Figure 2F).

We next investigated the postsynaptic responses to single light pulse stimulation of GRP neurons in GRPR_excit_ and GRPR_inhib_ neurons (Figures 2G and 2H). Unexpectedly, none of the 15 delayed firing GRPR_excit_ neurons that responded with an excitatory postsynaptic potential (EPSP) became sufficiently depolarized to fire action potentials. The EPSPs depolarized the recorded cells on average by only 9.0 ± 1.46 mV (n = 15). By contrast, in 7 out of the 11 tonic firing GRPR_inhib_ neurons, single brief light pulse stimulation of *Grp*-ChR2 neurons triggered action potentials with probabilities between 10% and 100% (Figure 2I). Five GRPR neurons exhibited firing patterns different from delayed or tonic firing. These neurons also failed to generate action potentials upon optogenetic GRP neuron stimulation.

### Repetitive Burst-like GRP Neuron Stimulation Renders Excitatory GRPR Neurons Spontaneously Active and Susceptible to Suprathreshold Excitation

**Figure 3:**
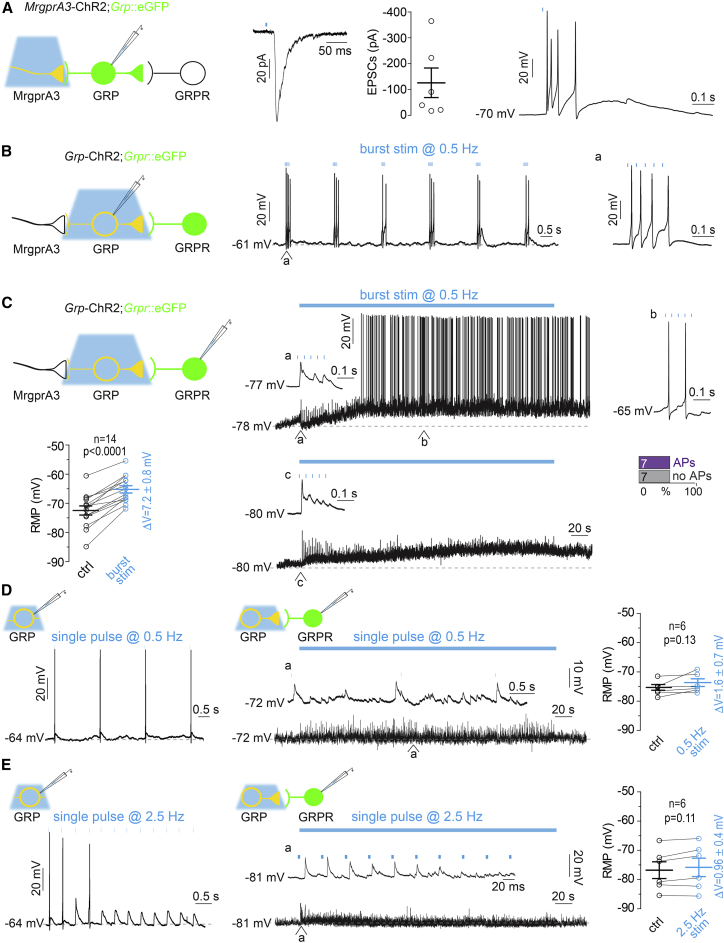
Suprathreshold Excitation of Delayed Firing GRPR_excit_ Neurons Requires Prolonged Burst-like Input from GRP Neurons (A) MrgprA3 fibers were stimulated with blue light (473 nm, 4 ms) and postsynaptic current or voltage responses were recorded from *Grp*-eGFP neurons. Left: experimental setup. Middle: EPSCs (average of five consecutive traces) and average EPSC amplitudes of 6 individual neurons. Left: burst firing in current-clamp in the same GRP neuron in response to the same blue light stimulation. (B) Repetitive light stimulation (five 4 ms pulses at 25 intra-burst frequency, repeatedly delivered at 0.5 Hz) of *Grp*-ChR2 neurons mimicked burst-like firing in response to input from MrgprA3 fibers. The first burst firing response is shown at higher resolution on the right (a). (C) Voltage responses recorded from two *Grpr*-eGFP neurons in response to repetitive burst-like blue light stimulation of *Grp*-ChR2 neurons (same stimulation as in B). (a)–(c) depict burst firing response at higher resolution at different time points of the experiment. Bar chart: incidence of GRPR neuron firing during sustained burst-like blue light stimulation (n = 14 cells from 13 animals). Paired plot: RMP before (black) and after 5 min of repetitive burst-like light stimulation (blue) (n = 14). Two-tailed paired t test, p < 0.0001. For the time course of changes in RMP and the incidence of action potentials (APs) during burst stimulation see Figure S4. (D and E) Same as (B) and (C) but repetitive single presynaptic light stimulations at 0.5 Hz (D) (n = 6 cells from 3 animals; two-tailed paired t test, p = 0.13) or 2.5 Hz (E) (n = 6 cells from 4 animals; two-tailed paired t test, p = 0.11). All error bars indicate SEM.

The above results prompted us to question whether the single brief light stimulation of the GRP neurons faithfully recapitulated the activity of GRP neurons evoked by input from peripheral pruritoceptors. To address this question, we used transgenic mice that express the cre recombinase specifically in peripheral pruritoceptors under the transcriptional control of the mas-related G-protein-coupled receptor A3 (*MrgprA3*::cre mice; Han et al., 2013). We prepared spinal cord slices from *MrgprA3*::cre;Ai32;*Grp*::eGFP (short, *MrgprA3*-ChR2;*Grp*::eGFP) mice and recorded postsynaptic responses from GRP neurons (Figure 3A). EPSCs were evoked by brief (4 ms) optogenetic stimulation of MrgprA3-positive pruritoceptors. Six out of 28 GRP neurons responded with EPSCs with average amplitudes of −126 ± 57 pA. When we switched to current-clamp mode, a single stimulation of MrgprA3 fibers triggered bursts of 2–5 action potentials in 4 out of the 5 GRP neurons. This burst-like firing corresponded well with the initial burst firing elicited by depolarizing current injection (cf. Figure 1). We decided to mimic this firing pattern in our subsequent experiments on GRP to GRPR neuron synaptic transmission through temporally patterned optogenetic stimulation. As most forms of itch involve a prolonged presence of the pruritic stimulus and sustained activation of pruritoceptors, we first tested whether GRP neurons would be able to sustain repeated burst-like activity over prolonged periods of time (Figure 3B). We found that GRP neurons were able to follow light-evoked burst-like stimulation with inter-burst intervals down to 2 s (0.5 Hz). Since pruritoceptors typically fire at similarly low rates (Ma et al., 2012, Schmelz et al., 1997), we considered that this pattern of GRP neuron stimulation would likely mimic the *in vivo* situation during ongoing pruritic stimulation. We then tested whether this prolonged burst-like stimulation of GRP neurons would be sufficient to render GRPR_excit_ neurons susceptible to suprathreshold activation (Figure 3C). All 14 GRPR neurons responded with a progressive slow depolarization that built up on average to 7.2 ± 0.8 mV over several minutes (n = 14; p < 0.0001, two-tailed paired t test). Four of the 14 GRPR_excit_ neurons showed action potentials already during the first series of burst stimulations. An additional 3 GRPR_excit_ neurons started to fire action potentials during continued *Grp*-ChR2 neurons stimulation. Six out of 11 neurons fired action potentials correlated with the blue light stimulation (for a time course of light triggered and spontaneous action potentials see Figure S3). Seven of the 14 GRPR neurons recorded also became spontaneously active, i.e., they fired action potentials uncorrelated with the optogenetic stimulation. Three out of the 14 neurons did not receive fast glutamatergic synaptic input from *Grp*-ChR2 neurons but still responded with a progressive depolarization and one of them also became spontaneously active. Depolarization and spontaneous activity persisted for minutes beyond the termination of synaptic stimulation. When GRP neurons were repetitively stimulated with single light stimuli (instead of bursts) at 0.5 Hz, depolarization of GRPR neurons amounted only to 1.6 ± 0.9 mV (n = 6; p = 0.13, two-tailed paired t test) and EPSPs remained subthreshold (Figure 3D). We also tried higher stimulation frequencies (2.5 Hz), i.e., applied the same number of light pulses as with the burst-like stimulation but separated at regular intervals. *Grp*-ChR2 neurons were not able to follow this stimulation for more than a few seconds (Figure 3E). Accordingly, GRPR_excit_ failed to exhibit a significant depolarization (0.96 ± 0.49 mV, n = 6; p = 0.11, two-tailed paired t test) and did not fire action potentials.

### Progressive Depolarization Depends on GRPR but Not Glutamate Receptor Signaling

**Figure 4:**
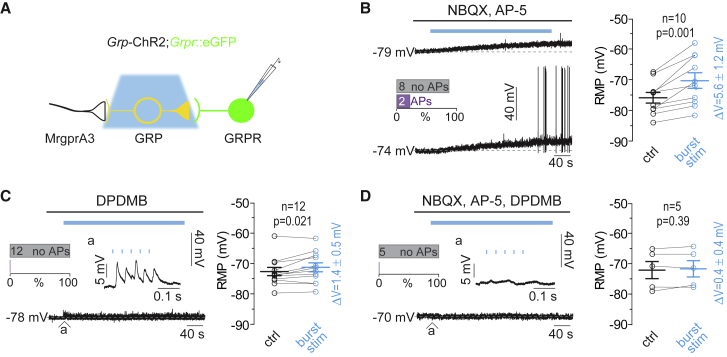
Suprathreshold Activation of Delayed Firing GRPR_excit_ Neurons during Repetitive Burst-like Stimulation of GRP Neurons Depends on GRP Release (A) Experimental setup. *Grp*-ChR2 neurons were excited with repetitive burst-like blue light stimuli and targeted current-clamp recordings were made from delayed firing GRPR_excit_ neurons. (B) Left: representative voltage traces in GRPR_excit_ neurons during 5 min burst-like light stimulation of GRP neurons (blue line) in the presence of NBQX (20 μM) and AP-5 (50 μM). Inset: incidence of GRPR_excit_ neuron firing during burst-like light stimulation (n = 10 cells from 7 mice). Right: paired plot showing RMP before (black) and 5 min after (blue) repetitive burst-like light stimulation. Circles are individual cells (n = 10 from 7 mice). Two-tailed paired t test, p = 0.001. (C) Left: same as (B) but in the presence of the GRPR blocker DPDMB (1 μM). Paired two-tailed t test, p = 0.021. (D) Same (B) but in the combined presence of NBQX, AP-5, and DPDMB. Two-tailed paired t test, p = 0.39. All error bars indicate SEM.

The observed progressive increase in excitability might be a consequence of GRP release from the GRP neurons. However, glutamate receptor-dependent plasticity constitutes an alternative mechanism. We therefore repeated the above experiment in the presence of the AMPA and NMDA receptor antagonists NBQX and AP-5. Mean progressive depolarization remained virtually unchanged (5.6 ± 1.2 mV; n = 10; p = 0.001, two-tailed paired t test), and some of the neurons started firing action potentials even in the absence of fast (phasic) glutamatergic input (Figures 4A and 4B). When we blocked GRP signaling with the peptide GRPR antagonist D-Phe^6^,Leu-NHEt^13^,des-Met^14^)-bombesin (6–14) (DPDMB, 1 μM), the progressive depolarization was nearly abolished (1.4 mV ± 0.5 mV; n = 12; p = 0.021, two-tailed paired t test), while light-evoked EPSPs remained unaltered (Figure 4C). In the combined presence of AMPA, NMDA, and GRPR antagonists, both progressive depolarization (0.4 mV ± 0.4 mV; n = 5; p = 0.39, two-tailed paired t test) and EPSPs were no longer detected (Figure 4D).

**Figure 5:**
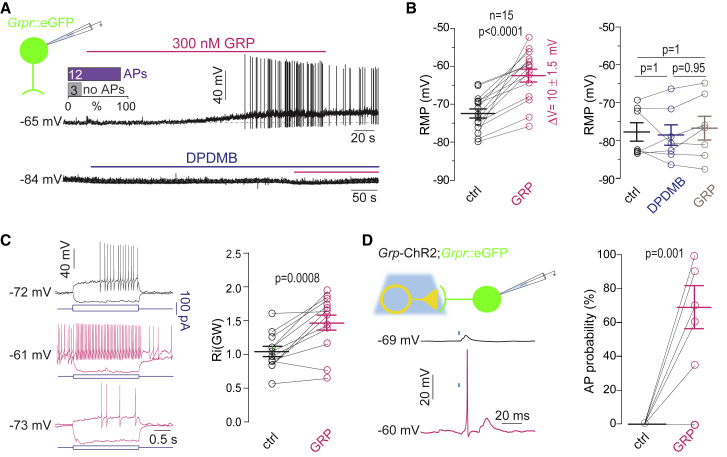
Exogenous GRP Application Mimics Effects of Repetitive Burst-like Stimulation of GRP Neurons (A) Voltage responses elicited by exogenous GRP (5 min, 300 nM) were recorded from delayed firing GRPR_excit_ neurons. Bar chart: incidence of GRPR_excit_ neurons that start firing action potentials during GRP application (n = 15 from 10 mice). DPDMB (1 μM, ≥15 min, blue bar) prevented GRP-mediated depolarization and action potential firing. (B) Left: paired plot showing RMP values before (black) and after GRP application (magenta, n = 15 from 10 mice). Two-tailed, paired t test. p < 0.0001. Right: same as left but with DPDMB applied before GRP (n = 7 cells from 3 mice). Repeated-measures ANOVA, F (2, 12) = 0.54, p = 0.59, followed by Bonferroni post hoc test. (C) Sample voltage traces during somatic injection of hyperpolarizing or depolarizing current steps (blue) in control condition (black) and in the presence of GRP (magenta). Bottom traces were recorded in the continuous presence of GRP but after repolarization to the RMP measured before GRP application. Delayed firing was recovered in all 5 neurons. Right: paired plot showing input resistance (R_i_) before (black) and after GRP application (magenta, n = 12 cells from 8 animals). Two-tailed, paired t test, p = 0.0008. (D) Voltage responses recorded from GRPR_excit_ neurons in response to stimulation of *Grp*-ChR2 neurons with 4 ms blue light pulses. In the absence of GRP, only subthreshold EPSPs were recoded (black trace). After 5 min of exposure to GRP, 7 out of 8 *Grpr*-eGFP fired action potentials upon blue light stimulation of *Grp*-ChR2 neurons (magenta trace). Paired plot: action potential probability before (black) and during GRP application (magenta, n = 8 cells from 5 mice). Two-tailed, paired t test, p = 0.001. All error bars indicate SEM.

Accordingly, exogenous GRP application mimicked the effects of sustained burst-like stimulation of *Grp*-ChR2 neurons (Figure 5A). Superfusion of the slices with 300 nM GRP induced a progressive depolarization of GRPR_excit_ neurons by 10.0 ± 1.5 mV (n = 15; p < 0.0001, two-tailed paired t test) and led to the generation of action potentials in 12 out of the 15 recorded neurons (Figure 5B). GRP-mediated depolarization was completely prevented by preincubation with the GRPR antagonist DPDMB (1 μM). Other apparent effects of GRP on GRPR_excit_ neurons included a change in the firing pattern from delayed to tonic firing and an increase in the input resistance from 1.04 ± 0.08 GΩ to 1.46 ± 0.12 GΩ (n = 12; p = 0.0008, two-tailed paired t test). When we repolarized the recorded neurons to their RMP measured before GRP application, the firing pattern changed back to delayed firing in five out of five neurons, indicating that the effect of GRP was primarily due to its depolarizing action (Figure 5C), consistent with a previous study on unidentified dorsal horn neurons that showed that depolarization alone was sufficient to change firing patterns from delayed to tonic (Ruscheweyh and Sandkühler, 2002). GRP not only induced spontaneous action potential firing but also rendered 7 out of 8 recorded GRPR_excit_ neurons susceptible to suprathreshold excitation by single GRP-ChR2 neuron action potentials (Figure 5D). Tonic firing GRPR_inhib_ neurons did not significantly depolarize upon either superfusion with GRP or repetitive burst-like stimulation of *Grp*-ChR2 neurons (Figures S4A and S4B).

### Block of Kir2-like Potassium Channels and a Subsequent Reduction in A-Type Potassium Currents Mediate GRP-Induced Depolarization of GRPR Neurons

**Figure 6:**
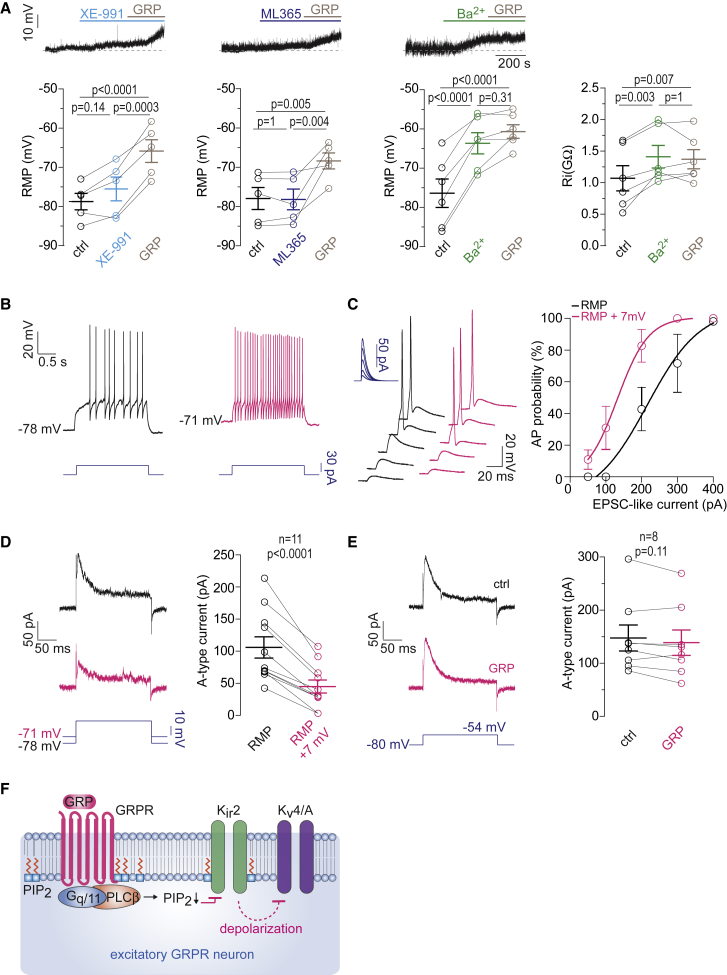
Downstream Signaling of GRPRs (A) Effects of XE-991 (10 μM, ≥6 min, light-blue bar, n = 5 from 3 mice), ML365 (10 μM, ≥ 6 min, blue bar, n = 5 from 4 mice), and Ba^2+^ (200 μM, ≥ 6 min, green bar, n = 6 from 3 mice) on the RMP of GRPR_excit_ neurons and on GRP-mediated depolarization (300 nM). Repeated-measurements one-way ANOVA, followed by Bonferroni post hoc tests, F(2,8) = 47.2 (XE-991), 14.4 (ML365), F(2,10) = 49.4 (Ba^2+^). p < 0.0001 (XE-991), p = 0.022 (ML365), p < 0.0001 (Ba^2+^). Right: changes in R_i_ induced by Ba^2+^ (200 μM) and GRP (300 nM). Repeated-measurements one-way ANOVA, followed by Bonferroni post hoc tests, F(2,17) = 12.7, p = 0.0018. (B and C) Depolarization by 7 mV of the RMP changed delayed firing into tonic-like firing (B) and increased action potentials probability in response to somatic EPSC-like current injections (C). Left: voltage trace examples evoked by EPSC-like current injections of increasing amplitude (50–400 pA). Right: stimulus response curves (n = 11 from 3 mice) fitted to the Boltzmann equation. (D) A 7 mV depolarization of the RMP reduced A-type potassium current amplitudes in GRPR_excit_ neurons by 61.9% ± 5.3% (n = 11, p < 0.0001, two-tailed, paired t test). (E) GRP (300 nM) had no effects on the A-type potassium current amplitude when the membrane potential was kept constant (7.0% ± 3.5%, n = 8 from 3 mice; p = 0.11; two-tailed, paired t test). (F) Schematic illustration of the intracellular signaling events triggered by GRPR activation in GRPR_excit_ neurons.

We next addressed the GRP signaling mechanisms that increase GRPR neuron excitability. GRP-mediated depolarization of GRPR neurons was accompanied by a 40% increase in membrane input resistance (R_i_) indicating that it was due to the closure of an outward conductance, presumably carried by potassium channels. GRPR typically signals via G proteins of the Gα_q_ family (Offermanns et al., 1994, Zachary et al., 1986). These G proteins inhibit different tonically active potassium channels, including members of the potassium channel families Kv7 (KCNQ2/3, also known as M-type currents) (Brown and Passmore, 2009), Kir2 (Hermes et al., 2013), and TASK-1/3 (KCNK-3 and KCNK-9) (Wilke et al., 2014). We used XE-991 (10 μM) (Tirko et al., 2018), ML365 (10 μM) (Zou et al., 2010), and low concentration Ba^2+^ (200 μM) (Li et al., 2013) to respectively block Kv7, TASK, and Kir2 channels and to test whether they would depolarize GRPR_excit_ neurons and occlude further depolarization by GRP (300 nM) (Figure 6A). Of these three blockers, only Ba^2+^ induced a significant depolarization (by 12.7 ± 1.4 mV, n = 6, p < 0.0001; repeated-measures one-way ANOVA) and prevented further depolarization by GRP (by 2.9 ± 1.4mV, n = 6, p = 0.31; repeated-measures one-way ANOVA). Like GRP, Ba^2+^ caused a significant increase in R_i_. In addition, it prevented further increases in R_i_ by GRP, suggesting that Ba^2+^-sensitive Kir2-like channels mediate GRP-induced depolarization.

We then tested whether a 7 mV depolarizing shift of the RMP (equivalent to the average depolarization induced by repetitive burst-like synaptic stimulation of GRPR neurons, see also Figure 3C) would replicate the changes in GRPR_excit_ neuron excitability observed with repetitive burst-like synaptic stimulation. This depolarization changed the firing pattern of GRPR_excit_ neurons from delayed to tonic-like firing (Figure 6B). Effects on activation of these neurons by excitatory synaptic input were tested with somatic current injections (50–400 pA amplitudes) that followed the time course of EPSCs measured in GRPR neurons (rise time of 0.49 ms, decay time of 4.28 ms). Depolarization of the RMP by 7 mV shifted the stimulus response curve to the left by 84 pA (Figure 6C).

Previous work has attributed a delayed firing to the presence of A-type potassium currents and showed that inhibition of these currents in dorsal horn neurons induces a switch from delayed to tonic firing (Ruscheweyh and Sandkühler, 2002). Because A-type currents undergo pronounced voltage-dependent inactivation, we tested whether the depolarization observed in GRPR_excit_ neurons with GRP application or with burst-like synaptic stimulation would reduce A-type currents. The 7 mV-depolarization of the RMP reduced the amplitude of A-type potassium currents by 61.9% ± 5.3% (n = 11, p < 0.0001, two-sided paired t test) (Figure 6D). Because A-type potassium channels are not only inactivated by prolonged depolarization but also inhibited by phosphorylation via extracellular signal-regulated kinase (Erk) (Hu et al., 2003), which can be initiated by Gα_q/11_-dependent signaling (Wagner et al., 2010), we tested whether the A-type potassium currents in GRPR neurons are also directly modulated by GRP. Under voltage-clamp conditions, A-type potassium currents were not changed by GRP (300 nM) (Figure 6E). Figure 6F summarizes this signaling cascade.

### Itch Behavior Elicited *In Vivo* by Repetitive Optogenetic Stimulation of Spinal GRP Neurons

**Figure 7:**
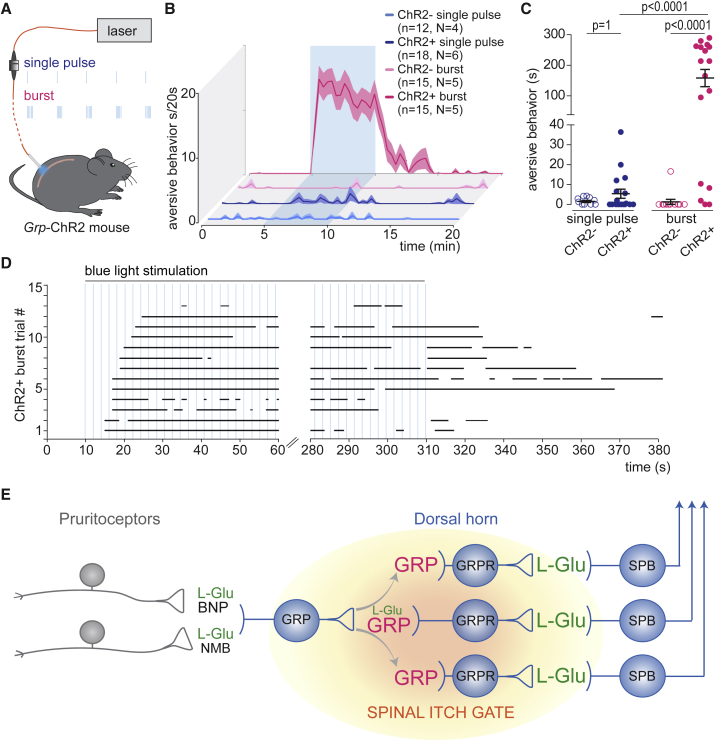
*In Vivo* Optogenetic Stimulation of GRP Neurons Requires Burst Stimulation Paradigms to Elicit Aversive Behavior (A) Experimental setup of *in vivo* optogenetic activation of spinal GRP neurons with single 4 ms pulses (at 0.5 Hz; blue) or burst stimulation (5 pulses at 25 Hz intra-burst frequency and repeated at 0.5 Hz; magenta). (B) Waterfall plot showing aversive behavior during unilateral optogenetic single pulse (4 ms) or burst-like (bursts of 5 pulses at 25 Hz with 0.5 Hz repetition rate; magenta) stimulation of GRP neurons in *Grp*-ChR2 mice (ChR2^+^) and Grp::cre^–^;ChR2 mice (ChR2^–^, n, number of trials; N, number of mice). Shaded lines represent mean ± SEM. (C) Categorical dot plots showing population data of aversive behavior during stimulation. Circles are individual trials. Error bars indicate mean ± SEM. Two-way ANOVA, followed by Bonferroni post hoc tests, F(1,56) = 39.31, p < 0.0001. (D) Time course of onset and cessation of light-evoked aversive behavior (black lines; n = 15 individual trials) in the five *Grp*-ChR2 mice. In two trials, blue light stimulation did not trigger aversive behaviors. (E) Strategic location of the GRP-GRPR neuron synapse in the spinal itch pathway. Synaptically released GRP is essential for the suprathreshold activation of GRPR neurons by glutamatergic input and induces spontaneous activity. GRP acts not only on synaptically connected neurons but also depolarizes GRPR neurons not directly connected via so-called volume transmission. SPB, spinoparabrachial projection neurons; BNP, B-type natriuretic peptide B; NMB, neuromedin B.

The above results obtained in spinal cord slices provide strong support for a critical contribution of GRP signaling to effective communication between GRP and GRPR neurons. They indicate that a continuous burst-like discharge activity in GRP neurons is needed to evoke sufficient GRP release, which allows action potential generation in GRPR_excit_ neurons and subsequently the spinal relay of pruritoceptive information. It is tempting to speculate that this particular dependence on GRP may underlie the rather slow *in vivo* onset and offset of itch. In order to provide further support for this idea, we performed optogenetic experiments *in vivo*. To this end, we chronically implanted GRP-ChR2 mice with fiber optics directed toward the right dorsal horn surface of the lumbar spinal cord segments L4/L5 (Bonin et al., 2016, Christensen et al., 2016). We then stimulated the GRP-ChR2 neurons with brief (4 ms) pulses of blue light and monitored aversive behavior (Figure 7A). Similar to what we had done in spinal cord slices, we compared single light stimulation repeated at a frequency of 0.5 Hz with burst-like light stimulation (bursts of 5 pulses of 4 ms duration each, applied at an intra-burst frequency of 25 Hz and repeated every 2 s). Significant behavioral changes were observed in 13 out of 15 trials performed in 5 GRP-ChR2 mice during burst-like light stimulation of GRP-ChR2 neurons (Figures 7B and 7C). By contrast, no behavioral changes occurred upon low-frequency stimulation with single light pulses (delivered every 2 s, n = 6 mice), and no changes were observed in ChR2-negative (GRP-cre^–^;Ai32) mice after either stimulation paradigm (n = 4 and n = 5, for single pulse and burst-like light stimulation). In line with the time course of action potential firing of GRPR_excit_ neurons observed in slices, the onset of aversive behavior also occurred only with a certain delay of 5–25 s, corresponding to the 3rd to 12th burst (Figure 7D). Even more striking was that aversive behavior outlasted the cessation of light stimulation by several minutes. This persistence of behavioral responses resembles the time course of GRPR neuron action potential firing observed in slices, which also extended for several minutes beyond the termination of light stimulation. These similarities are remarkable in particular as light-evoked activity of GRP neurons *in vivo* would add to any spontaneous ongoing activity.

## Discussion

The present study was triggered by the questions why itch, in contrast to pain, depends critically on neuropeptide signaling, specifically on signaling via the neuropeptide GRP, and whether this neuropeptide signaling might contribute to the low temporal resolution of itch sensations. We have focused our efforts on the relay of spinal itch signals from second-order GRP to third-order GRPR neurons. We found that, although both neuron types were coupled via monosynaptic glutamatergic connections, single presynaptic action potentials in GRP neurons were not sufficient to evoke suprathreshold postsynaptic excitation of GRPR_excit_ neurons; i.e., they were unable to drive action potential firing in GRPR_excit_ neurons. Only when GRP neurons were driven to fire in bursts, suprathreshold activation was achieved. Furthermore, in many of the GRPR_excit_ neurons prolonged burst activity was required to elicit action potential firing, which then persisted for minutes beyond the termination of burst stimulation. A similar dependence on presynaptic burst activity and similarly delayed onset and offset of itch responses were observed *in vivo* when spinal GRP neurons were optogenetically stimulated, indicating that it is this dependence of GRPR_excit_ neuron activation on conditioning depolarization that makes itch critically dependent on GRP signaling. About half of the recorded GRPR_excit_ neurons not only became responsive to excitatory synaptic input but also became spontaneously active upon repeated burst-like GRP neuron input or exposure to exogenous GRP. Appearance of spontaneous activity indicates that GRP did not only prime GRPR_excit_ neurons for suprathreshold activation but was also able to provoke spontaneous activity. The GRPR_inhib_ neurons differ from their excitatory cousins not only in their firing pattern and neurochemistry but also in their immediate excitability by input from GRP neurons. The function of these neurons is currently unknown but they might potentially be elements of a pain inhibitory circuit initiated from pruritoceptive neurons.

### Presynaptic Features Supporting GRP Release

Excitatory GRPR neurons did not fire action potentials after single or regularly spaced repetitive single light-pulse synaptic stimulations; instead, they became activated only after burst-like stimulation of GRP neurons. Repetitive burst firing induces larger and more sustained rises in presynaptic Ca^2+^ to enable efficient neuropeptide release (Bruns and Jahn, 1995, Leenders et al., 1999, van den Pol, 2012). Interestingly, it has been shown that release of vasopressin arginine peptide from the neurohypophysis is optimally triggered by short phasic burst stimulation protocols, which lead to gradual built-up of residual Ca^2+^ levels in the presynaptic terminal (Muschol and Salzberg, 2000). In fact, although amino acid and neuropeptide transmitters are present in the same axon terminals, they are stored in different classes of vesicles, with neuropeptides being released from so-called dense core vesicles (Johansson et al., 1980, Torrealba and Carrasco, 2004). Their location in the presynaptic terminal is more diffuse and more distant from the voltage-gated Ca^2+^ channels in the active release zones, which explains why peptide release requires more sustained and widespread Ca^2+^ signals (Bruns and Jahn, 1995). The initial burst-firing pattern that is found in the vast majority of GRP neurons promotes strong rises in intracellular Ca^2+^ and thus fosters neuropeptide release. In addition, their relatively broad action potentials further support intracellular Ca^2+^ rises (Bean, 2007).

Co-release of fast amino acid transmitters with a peptide transmitter is a widespread phenomenon in the mammalian CNS (Nusbaum et al., 2017). A large body of literature also describes slow depolarization or hyperpolarization (depending on the downstream signaling cascades) induced by exogenous application of neuropeptides or by repetitive presynaptic stimulation (Strand, 1999, van den Pol, 2012). However, few, if any, reports have shown such a critical dependence of suprathreshold postsynaptic excitation on co-release of a neuropeptide.

A typical feature of neuropeptide signaling is volume transmission, which, by its underlying mechanisms, occurs rather slowly, on a timescale of seconds to minutes, and typically reaches well beyond the structural extent of synaptic connections (Fuxe et al., 2007). In agreement with this concept, our experiments showed that repetitive burst-like stimulation induced depolarization not only in GRPR neurons with direct glutamatergic input from GRP neurons but also in neurons that lacked such direct input. This spatially extended signaling may contribute to our rather poor ability to localize itch stimuli.

### Downstream GRPR Signaling Cascades

In agreement with earlier studies that analyzed the effects of exogenously applied GRP in the spinal dorsal horn (Aresh et al., 2017, Koga et al., 2011, Kusube et al., 2016), we found that GRP-mediated depolarization of GRPR neurons was accompanied by a 40% increase in R_i_. Our experiments with several potassium channel blockers revealed that the increase in R_i_ and the subsequent depolarization resulted from the inhibition of tonically active Ba^2+^-sensitive potassium currents, likely mediated by inwardly rectifying potassium channels of the Kir2 family, thereby recapitulating GRP actions in thalamic neurons (Hermes et al., 2013). Members of the Kir2 family, in particular, Kir2.2 channels, are extensively expressed in the superficial dorsal horn (Prüss et al., 2005) providing further support for their involvement. Activity of Kir2 channels requires the presence of phosphatidylinositol-4,5-bisphosphate (PIP2) in the cell membrane (Lee et al., 2016), which gets depleted upon activation of phospholipase Cβ (PLCβ). The susceptibility of PLCβ to activation by Gα_q/11_ links this pathway to GRPR activation. Our results are also consistent with an additional protein kinase-dependent regulation of Kir2 channels. Other potassium channels that are inhibited by Gα_q/11_-dependent signaling, such as TASK1/3 and KCNQ2/3 channels (Brown and Passmore, 2009, Suh et al., 2004, Wilke et al., 2014),are also expressed in the spinal dorsal horn (Gabriel et al., 2002, Prüss et al., 2005, Talley et al., 2001), but did apparently not contribute.

Signaling steps subsequent to inhibition of Kir2 channels probably involve depolarization-induced inactivation of the A-type potassium currents. These currents underlie the relatively long delay with which action potentials occur after a depolarizing current injection in delayed firing dorsal horn neurons (Ruscheweyh and Sandkühler, 2002, Yoshimura and Jessell, 1989). Their pharmacological inhibition with 4-aminopyridine (Ruscheweyh and Sandkühler, 2002, Yoshimura and Jessell, 1989) or genetic ablation of the underlying Kv4.2 channels (Hu et al., 2006) increase the excitability of dorsal horn neurons. A-type potassium channels, in particular, Kv4.2 channels, are not only inactivated by prolonged depolarization but also by phosphorylation via Gα_q/11_-dependent extracellular signal regulated kinase (Erk) (Hu et al., 2003, Wagner et al., 2010). Furthermore, it has been suggested that GRPR-mediated itch responses occur through downstream activation of the phosphoinositid 3-kinase γ (PI3Kγ)/Akt pathway (Pereira et al., 2015). However, our results do not support a depolarization-independent effect of GRP on A-type currents.

### Features Underlying the Differential Susceptibility of Excitatory and Inhibitory GRPR Neurons to GRP Neuron Input

Differences in two biophysical characteristics between delayed firing GRPR_excit_ and tonic firing GRPR_inhib_ neurons may explain their different susceptibility to suprathreshold excitation by glutamatergic input from GRP neurons. First, the rheobase (i.e., the minimum depolarizing current sufficient to trigger an action potential) is 3.5 times higher in GRPR_excit_ versus GRPR_inhib_ neurons. However, the average GRP neuron-evoked EPSC in GRPR_excit_ neurons exceeded the rheobase by more than 4-fold, questioning whether the difference in the rheobase is the major determinant. A second potentially contributing factor are A-type potassium channels, which underlie the delayed firing pattern in excitatory dorsal horn neurons but are absent from inhibitory dorsal horn neurons (Ruscheweyh and Sandkühler, 2002). These A-type potassium currents become quickly activated upon depolarization and thereby effectively limit the depolarization of neurons by postsynaptic glutamatergic input. The relevance of this process for transmission across the GRP to GRPR neuron synapse is underscored by our observation that GRP neuron-evoked EPSPs depolarized GRPR_excit_ neurons on average by only 9 mV, i.e., to about −64 mV, at the peak of the EPSP. This value is far from the action potential threshold of GRPR_excit_ neurons (−41.5 mV, cf. Results and Table 1). It is hence most likely the A-type potassium currents that limit the susceptibility of the GRPR_excit_ neurons to suprathreshold activation by glutamatergic input from GRP neurons.

### Summary and Implications for the Systems Physiology of Itch

Our results identify a cellular and neurophysiological basis for the critical contribution of spinal GRP signaling to itch behaviors. On the cellular level, GRP inhibits a tonic outward conductance (likely Kir2 potassium current) in GRPR_excit_ neurons. This inhibition depolarizes GRPR_excit_ neurons, partially inactivates their A-type potassium currents and renders them more excitable to synaptic input and even spontaneously active (Figure 6F). On a circuit level, GRP-releasing and GRP-sensing (GRPR) neurons are placed at a particular strategic site between peripheral pruritoceptive input and spinoparabrachial output neurons (Figure 7E). While fast glutamatergic signaling is apparently sufficient for signal relay at the first and last synapse of this tri-synaptic pathway (Figure 3A; Aresh et al., 2017), the synapse between the GRP and GRPR neuron requires GRP released during repetitive burst-like presynaptic activity to open the spinal gate for itch signals. GRP signaling thus adds an additional level of sophistication to other already well-established control mechanisms of spinal itch transmission including fast inhibitory control via GABA and glycine receptors (Foster et al., 2015, Ralvenius et al., 2018, Ross et al., 2010) and via opioid peptide signaling (Huang et al., 2018, Kardon et al., 2014).

## STAR★Methods

### Key Resources Table

**Table:** 

REAGENT or RESOURCE	SOURCE	IDENTIFIER
**Antibodies**		

Rabbit anti-GFP	Molecular Probes	RRID: AB_221570
Guinea pig anti-Lmx1b	Dr Carmen Birchmeier (Müller et al., 2002)	N/A
Goat anti-Pax2	R and D Systems	RRID: AB_10889828
Rabbit anti-Tlx3	Dr Carmen Birchmeier (Müller et al., 2002)	N/A
Goat anti-tdTomato	SICGEN	RRID: AB_2722750
Guinea pig anti-VGluT2	Millipore	RRID: AB_2665454
Cyanine 3 Cy3-donkey anti-goat	Jackson ImmunoResearch	RRID: AB_2340413
Alexa Fluor 488-donkey anti-rabbit	Jackson ImmunoResearch	RRID: AB_2340619
Alexa Fluor 647-donkey anti-guinea pig	Jackson ImmunoResearch	RRID: AB_2340477
Biotin-donkey anti-Rabbit	Thermo Fisher Scientific	RRID: AB_228212
Alexa Fluor 488-Streptavidin	Jackson ImmunoResearch	RRID: AB_2337249

**Chemicals, Peptides, and Recombinant Proteins**		

NBQX: NBQX disodium salt	Biotrend	Cat# BN0608
GRP: Gastrin Releasing Peptide, human	Anaspect	Cat# AS-24214
DPDMB: (D-Phe^6^,Leu-NHEt^13^,des-Met^14^)-Bombesin (6-14) trifluoroacetate salt	Bachem	CAS# 124199-90-2; Product# 4030433; Cat# H-3042
TTX: TTX citrate	Tocris Bioscience	CAS# 18660-81-6; Cat# 1069
AP-5: D-AP5	Tocris Bioscience	CAS# 79055-68-8; Cat# 0106
XE-991: XE 991 dihydrochloride	Tocris Bioscience	CAS# 947914-18-3; Cat# 2000
ML365: ML365	Tocris Bioscience	CAS# 122955-13-9; Cat# 5337
Ba^2+^: Bariumchlorid-Dihydrat	MERCK	CAS# 10326-27-9; Cat# 101719
Biocytin	MERCK	CAS# 576-19-2

**Deposited Data**		

Raw and analyzed data	This paper	https://doi.org/10.17632/9p3tb2j2nf.1

**Experimental Models: Organisms/Strains**		

Mouse: Ai32: B6;129S-*Gt(ROSA)26Sor*^*tm32(CAG-COP4∗H134R/EYFP)Hze*^/J	The Jackson Laboratory	RRID: IMSR_JAX:012569
Mouse: Ai14: B6;129S6-*Gt(ROSA)26Sor*^*tm14(CAG-tdTomato)Hze*^/J	The Jackson Laboratory	RRID: IMSR_JAX:007914
Mouse: GRP::eGFP: STOCK Tg(Grp-EGFP)DV197Gsat/Mmucd	MMRRCC GENSAT	RRID: MMRRC_010444-UCD
Mouse: GRPR::eGFP: STOCK Tg(Grpr-EGFP)PZ62Gsat/Mmucd	MMRRCC GENSAT	RRID: MMRRC_036178-UCD
Mouse: GRP::cre: STOCK Tg(Grp-cre) KH288Gsat/Mmucd	MMRRCC GENSAT	RRID: MMRRC_031183-UCD
Mouse: MrgprA3::cre: *MrgprA3*^GFP-Cre^	Dr. Xinzhong Dong (Han et al., 2013)	N/A

**RNAscope *in situ* hybridization probes**		

RNAscope Probe- EGFP	Advanced Cell Diagnostics	Cat. No. 400281
RNAscope Probe- Mm-Grpr	Advanced Cell Diagnostics	Cat No. 317871
RNAscope Probe- Mm-Grpr-C2	Advanced Cell Diagnostics	Cat. No. 317871-C2
RNAscope Probe- Mm-Slc17a6-C2	Advanced Cell Diagnostics	Cat No. 319171-C2
RNAscope Probe- Mm-Slc32a1-C2	Advanced Cell Diagnostics	Cat No. 319191-C2
RNAscope Probe- EGFP-C3	Advanced Cell Diagnostics	Cat No. 400281-C3

**Software and Algorithms**		

Igor Pro 6.22A	Wavemetrics	https://www.wavemetrics.com/downloads/current
ImageJ	National Institutes of Health (NIH)	https://imagej.nih.gov/ij/download
Patchmaster, version 2x80	HEKA, Harvard Bioscience	http://www.heka.com/downloads/software/old/MacOS/OSX/Patchmaster%20family/2x80/
Prism 5	GraphPad	https://www.graphpad.com/scientific-software/prism
Live Acquisition Software v2.2.0	TILL Photonics	no longer distributed
ZEN 2011 (black edition)	Carl Zeiss	https://www.zeiss.com/microscopy/int/downloads
ZEN 2.3 (blue edition)	Carl Zeiss	https://www.zeiss.com/microscopy/int/downloads
LKTerm (version 1.1.0.0)	Loksoft	https://www.loksoft.ch/sites/downloads/dlTerminal.aspx

### Contact for Reagents and Resource Sharing

Further information and requests for resources and reagents should be directed to and will be fulfilled by the Lead Contact, H.U. Zeilhofer (zeilhofer@pharma.uzh.ch).

### Experimental Model and Subject Details

#### Mouse lines

BAC transgenic mouse lines used include *Grp*::eGFP, *Grpr*::eGFP, *Grp*::cre (all from GENSAT), and *MrgprA3*::cre mice (provided by Dr. Xinzhong Dong, Johns Hopkins University; Han et al., 2013). Cre lines were crossed with Ai32 (B6;129S-Gt(ROSA)26Sortm32(CAG-COP4^∗^H134R/EYFP)Hze/J) mice for optogenetic experiments and with Ai14 (B6.Cg-Gt(ROSA)26Sortm14(CAG-tdTomato)Hze/J) mice for immunostaining experiments. BAC transgenic mouse lines were maintained in the heterozygous state in the C57BL/6 genetic background. Animals used for *in vivo* optogenetic experiments (Figure 7) were single-housed after cannula implantation. All other experimental animals were kept group-housed under intermediate barrier conditions (https://www.jax.org/) and under a 12/12-hour light/dark cycle with *ad libitum* access to food and water. Experimental animals did not show any pathology and were drug or test naive before use in this study. Permission for all animal experiments was obtained from the Kanton of Zurich (licenses 031/2016 and 174/2016). All animal experiments complied with the relevant ethical regulations.

### Method Details

#### Slice preparation and electrophysiological recordings

Transverse spinal cord slices (400 μm thick) were prepared from 3 - 5 week-old mice of either sex. Slices were cut in ice-cold solution containing (in mM): 130 K-gluconate, 15 KCl, 0.05 EGTA, 20 HEPES, and 25 glucose (pH 7.4) (Dugué et al., 2005) using a vibrating blade microtome (D.S.K., microslicer DTK 1000). Slices were allowed to recover at 37°C for 15 min in a solution containing (in mM): 225 D-mannitol, 2.5 KCl, 1.25 NaH_2_PO_4_, 25 NaHCO_3_, 8 MgCl_2_, 0.8 CaCl_2_ and 25 glucose (pH 7.4), equilibrated with 95% O_2_, 5% CO_2_. Following recovery, slices were transferred and maintained in artificial cerebrospinal fluid (ACSF, 37°C) containing (in mM): 120 NaCl, 2.5 KCl, 1.25 NaH_2_PO_4_, 26 NaHCO_3_, 5 HEPES, 1 MgCl_2_, 2 CaCl_2_ and 14.6 glucose (pH 7.4), equilibrated with 95% O_2_, 5% CO_2_.

Targeted whole-cell patch-clamp recordings from *Grp*-eGFP and *Grpr*-eGFP neurons were performed at room temperature using epifluorescence for neuron identification followed by infrared gradient contrast for placing of the recording pipette. During recordings, slices were continuously superfused with ASCF at a rate of 1 - 2 mL min^-1^. Patch pipettes (borosilicate glass; 3.5 - 4.5 MΩ; Harvard Apparatus) were filled with intracellular solution containing (in mM): 130 K^+^ gluconate, 5 NaCl, 1 EGTA, 10 HEPES, 5 Mg-ATP, 0.5 Na-GTP (pH 7.35, 290 - 300 mosm l^-1^). Membrane potentials were corrected for the liquid junction potential of +15.2 mV.

The biophysical properties of GRP neurons were investigated in spinal cord slices prepared from *Grp*::eGFP mice. Biophysical properties of GRPR neurons and the effect of exogenous GRP on GRPR neurons were determined with targeted patch-clamp recordings in slices prepared from *Grpr*::eGFP and *Grp*-ChR2;*Grpr*::eGFP mice. Additional patch-clamp recordings were performed in slices prepared from *MrgprA3*-ChR2;*Grp*::eGFP and *Grp*-ChR2;*Grpr*::eGFP mice, as reported in the figures. Passive and active biophysical properties of GRP and GRPR neurons were examined in current-clamp mode. The membrane potential recorded after switching from voltage-clamp to current-clamp mode, was considered the RMP (RMP). Input resistance (R_input_) and membrane capacitance (C_m_) were determined through injection of hyperpolarizing current steps (2 s, −5 pA increments, delivered every 10 s). Action potential (AP) firing was evoked by depolarizing current steps of increasing magnitude (2 s, +10 pA increments, delivered every 10 s). Single AP properties (threshold, amplitude, width and afterhyperpolarization) were determined considering the first action potential at rheobase. AP firing patterns evoked by depolarizing current injection were classified according to previously published criteria (Abraira et al., 2017, Punnakkal et al., 2014). Briefly, delayed (D) firing neurons were characterized by a prominent delay between the onset of the depolarizing step and the AP discharge. Tonic (T) neurons were characterized by persistent APs discharge. Phasic (P) neurons featured a burst of action potentials at the rheobase that became persistent injecting current of higher magnitude. Neurons with a prominent gap between series of AP discharges were classified as gap (G) firing and neurons with a burst of action potentials at the beginning as initial bursting (Ib) neurons.

To activate ChR2 in acute slices, wide field illumination through a 40x water immersion objective (W Plan-Apochromat, Zeiss) was applied using a Polychrome V monochromator controlled using Live Acquisition Software v2.2.0 (TILL Photonics, Gräfelfing, Germany). GRP neurons or MrgprA3-expressing terminals were stimulated with pulses of blue light (473 ± 5 nm wavelength, 1.15 mW). After identification of GFP neurons we implemented a recovery period of 10 - 15 min before the patch-clamp recording to allow neurons to recover from blue light exposure. Latency, jitter and failure rate of synaptic responses were considered as criteria for monosynaptic connections. The latency was determined between light onset and the AP peak or the onset of the EPSC. We noted that light exposure followed the output trigger signal with a delay of 3.38 ms (attributable to electronic and mechanical delay). AP and EPSC latencies were corrected for this delay. The jitter was calculated as the standard deviation of the latency values of twenty consecutive EPSCs. Light-evoked EPSCs were recorded at a holding potential of −70 mV.

Access resistance was continuously monitored with short hyperpolarizing voltage steps. Recordings in which the access resistance changed by more than 20% during the experiment and cells with initial RMPs more depolarized than −55 mV were excluded from the analysis. Data were acquired using an EPC9 amplifier (HEKA Elektronik, Lambrecht, Germany) controlled with Patchmaster, version 2x80 acquisition software and sampled at 20 kHz. Data were analyzed using IGOR Pro 6.22A.

#### Immunohistochemistry and image analysis

Six to twelve week-old mice of either sex were anaesthetized with pentobarbital (160 mg kg^−1^, i.p.) before transcardiac perfusion with 20 mL of ice-cold ACSF followed by 100 mL of 4% ice-cold paraformaldehyde (in 0.1 M sodium phosphate buffer, pH 7.4). Spinal cords tissue was post-fixated for 2 h with 4% paraformaldehyde on ice, cryoprotected in 25% sucrose solution (in 0.1 M sodium phosphate buffer) overnight at 4°C, embedded in NEG50 frozen section medium (Richard-Allen Scientific) and stored at −80°C until use. The spinal cords were cut into 30 μm cryosections using Hyrax C60 cryostat (Carl Zeiss) and mounted onto Superfrost Plus microscope slides (Thermo Fisher Scientific). Spinal cord section were incubated at 4°C overnight in a primary antibody solution (PBS, 0.3% Triton X-100, 10% normal donkey serum) containing combinations of the following antibodies: rabbit anti-GFP (1:1000), guinea pig anti-Lmx1b (1:10,000), goat anti-Pax2 (1:200), goat anti-tdTomato (1:1000), guinea pig anti-vGluT2 (1:1000). Three washing steps of 5 min each in PBS were performed before incubating spinal cord sections with secondary antibodies (1:800) for 1h at room temperature in PBS supplemented with 0.3% Triton X-100. For details on the antibodies, see Key Resources Table. Immunostaining of synaptic contacts between GRP and GRPR neurons was performed on 40 μm thick free floating sections (cutting was performed using Hyrax KS 34 microtome, Carl Zeiss) and the sections were pretreated 3 times for 10 min with 50% ethanol (in ddH_2_O), washed two-times for 10 min in PBS and incubated with primary antibodies for 3 days. Images were taken with a LSM 710 or LSM 800 with Airyscan confocal microscopes (Carl Zeiss) controlled with ZEN 2011 (black edition) or ZEN 2.3 (blue edition) software, respectively, and using, respectively, an EC Plan-Neofluar 40x/1.30 oil-immersion objective or a Plan-Apochromat 40x/1.4 Oil DIC M27 oil-immersion objective. Z stack images of 8 optical sections and 1.5 μm step size were used for the analysis of fluorescence colocalization and to create maximum intensity projections images, whereas Z stack images of 32 optical sections and 0.2 μm step size were used for the analysis of synaptic contacts. Images were processed using ImageJ software. For quantification, 3 or 5 animals and three sections per animal were analyzed. Cell counting was performed using the ImageJ Cell Counter plug-in.

To correlate the firing patterns with either an excitatory or an inhibitory phenotype *Grpr*::eGFP neurons were filled during whole-cell recording with a K^+^-gluconate based internal solution containing biocytin (1.5 mg/ml). Slices were transferred to a 4% paraformaldehyde fixative solution and incubated for 1 h at 4°C. Afterward they were cryo-protected overnight in 20% sucrose in PB before embedding and freezing in NEG50 for sectioning. Embedded sections were re-sectioned at 25μm and mounted on Superfrost Plus microscope slides. Antibody incubation with goat anti-Pax2 and rabbit anti-Tlx3 was carried out as described above. Streptavidin-488 conjugate was applied together with the secondary antibodies at a 1:500 dilution.

#### *In situ* hybridization

Spinal cords used for *in situ* hybridization were dissected from 6 - 10 week-old mice of either sex in ice-cold ACSF and immediately frozen in 1.5 mL Eppendorf tubes immersed in liquid nitrogen. Tissue was cut into 20 μm cryosections, mounted onto Superfrost Plus microscope slides (Thermo Fisher Scientific) and hybridized following RNAscope Assay guidelines (Advanced Cell Diagnostics, Newark, CA, USA), using probes designed for RNAscope Fluorescent Multiplex *in situ* hybridization listed in the Key Resources Table.

#### Fiber optic cannula implantation

Six to eight week-old male *Grp-*ChR2 (*Grp*::cre;Ai32 double transgenic) mice were implanted with fiber optic cannulas as was described previously (Bonin et al., 2016, Christensen et al., 2016). Control experiments were performed in *Grp*::cre^-^;Ai32 mice. Ceramic ferrules measuring ⌀ 1.25 mm (Thorlabs) were mounted with appropriate multimode optical fiber and trimmed < 1 mm at the edges. Mice were anesthetized with 2 - 5% isofluorane and maintained on a motorized stereotactic frame until end of surgical procedure in 1 - 2% isofluorane anesthesia. The fur over the back of the mice was shaved and an incision was made on the skin to expose the vertebral column. Incisions were made on the muscles lateral to the tendons spanning either sides of the T13 vertebral disc. The vertebral column was clamped with spinal adaptors and the T13 vertebral disc was exposed. The tissues covering the spinous and transverse processes of the disc were removed using forceps, and a hole was drilled on the caudal-transverse process approximately 2 mm from the midline to expose the L4 - L5 spinal cord segment. A rubber aspirator was used to dry the vertebral disc. Collagen strips (Lyostypt, B. Braun) were used to minimize bleeding. Small amounts of base-coat (One Coat 7 Universal, Coltene) were carefully applied to the cannula’s concave end and around the drilled hole on the spinous process as well as the rostro-caudal transverse processes. The coating was cured with UV light to provide a steady base for adherence. The fiber-optic cannula was inserted into the drilled hole. A layer of dental cement (Synergy D6 Flow, Coltene) was applied around the cannula over the base-coat, cured with UV-light for 20 s, and upon hardening, a second layer of dental cement was applied and cured to firmly secure the cannula to the vertebral disc. The muscles around the vertebral column were then sutured using absorbable sutures (Safil 5-0, B. Braun) and the skin was sutured with non-absorbable sutures (Dafilon 6-0, B. Braun). The mice were allowed to recover on a heat pad. Behavior experiments started 48 h after surgery.

#### *In vivo* optogenetic stimulation and behavior

To measure optogenetically-evoked behavior, mice were placed in cylinders and the fiber-optic cannula was connected via a mating sleeve to a 400 μm, 0.39 NA multimode fiber-optic patch cable (Thorlabs, Inc) that could rotate to allow free movement of the mouse. After coupling of the cannula to the patch cable under brief isoflurane anesthesia, the animals were habituated for 30 min. Light was delivered to the spinal cord from a 473 nm laser (Laserglow Technologies) connected to the fiber-optic patch cable. Timing of light stimulation was controlled by custom-written scripts in LKTerm software (Loksoft). Aversive behavior (biting or scratching) was recorded for 5 min prior light stimulation. This was followed by periods of 5 min during which the mouse was stimulated with single light pulses (4 ms, 473 nm, 0.5 Hz) or with bursts of light pulses (bursts of 5 pulses of 4 ms duration each at 25 Hz, repeated at a frequency of 0.5 Hz) and for a 10 min post-stimulus period. The experimenters were blind to the genotype of the mice. Analysis was done offline in slow motion, at 1/4 normal speed. Aversive behavior elicited by the optogenetic stimulation consisted mainly of fast small amplitude movements of the head directed to the ipsilateral hindlimbs. This behavior is considered a typical itch response (LaMotte et al., 2011). Each animal was tested over three trials run on consecutive days. After the experiments, mice were sacrificed and the correct placement of the fiber-optic cannulas was verified. The fiber-optic cannulae were then removed and coupled to the patch cable for measurement of output light intensities *ex-vivo*. Light intensities ranged from 0.7 - 2.0 mW (on average 1.4 ± 0.1 mW).

#### Drugs and Chemicals

NBQX (20 μM, Bio Trend), XE-991 (10 μM) and ML 365 (10 μM) were dissolved in DMSO (end concentration 0.02%). GRP (final concentration 300 nM, Anaspect), DPDMB (D-Phe^6^,Leu-NHEt^13^,des-Met^14^)-bombesin (6-14) trifluoroacetate salt (1 μM), TTX citrate (1 μM), AP-5 (50 μM, Tocris) and BaCl_2_ were dissolved in water.

### Quantitation and Statistical Analysis

All data are given as mean ± standard error of mean (sem). The number of animals used per experiment is described in the figure legends. Statistical comparisons were made using paired t test to compare measurement from two groups. Where independent multiple groups were compared in a single condition, one-way ANOVA followed by Bonferroni multiple comparisons *post-test* was used. Where related multiple groups were compared in a single condition, repeated-measurements ANOVA followed by post hoc Bonferroni correction was used. Where multiple groups tested with multiple conditions were compared, a two-way ANOVA followed by post hoc Bonferroni correction was applied. Where appropriate, a D’Agostino & Pearson normality test was conducted to assess if the data fit a normal distribution. All statistics were performed using Prism 5 (GraphPad, La Jolla, CA).

### Data and Software Availability

The raw data obtained in the study and the settings file used to control the light source in the *in vivo* optogenetic experiments are available at: https://doi.org/10.17632/9p3tb2j2nf.1.

## Supplemental Information

Document S1. Figures S1–S4

Document S2. Article plus Supplemental Information
