# Supplementary material for: How Gastrin-Releasing Peptide Opens the Spinal Gate for Itch
Source: Neuron. 2019 Jul 3;103(1):102–117.e5. doi: 10.1016/j.neuron.2019.04.022 (PMC6616317; doi:10.1016/j.neuron.2019.04.022)
Supplement: Document S1. Figures S1–S4 [file mmc1.pdf]

Neuron, Volume *103*

Supplemental Information

How Gastrin-Releasing Peptide

Opens the Spinal Gate for Itch

Martina Pagani, Gioele W. Albisetti, Nandhini Sivakumar, Hendrik Wildner, Mirko Santello, Helge C. Johannssen, and Hanns Ulrich Zeilhofer

Supplemental Information

How gastrin-releasing peptide opens the spinal gate for itch

[1]Martina Pagani, [1]Gioele W. Albisetti, [1]Nandhini Shivakumar, [1]Hendrik Wildner, [1]Mirko Santello, [1]Helge C. Johannssen, [1,2,3,4,]*Hanns Ulrich Zeilhofer

[1]Institute of Pharmacology and Toxicology, University of Zurich, Winterthurerstrasse 190, CH-8057 Zurich, Switzerland

[2]Neuroscience Center Zurich, Winterthurerstrasse 190, CH-8057 Zurich, Switzerland

[3]Drug Discovery Network Zurich, Winterthurerstrasse 190, CH-8057 Zurich, Switzerland

[4]Institute of Pharmaceutical Sciences, Swiss Federal Institute of Technology (ETH) Zurich, Vladimir-Prelog-Weg 1-5/10, CH-8090 Zurich, Switzerland

* lead contact: Dr. H.U. Zeilhofer, Institute of Pharmacology and Toxicology, University of Zurich, Winterthurerstrasse 190, CH-8057 Zurich, Switzerland.

Phone: +41 44 63 55912, FAX: +41 44 63 55988, Email: zeilhofer@pharma.uzh.ch

Supplemental figures

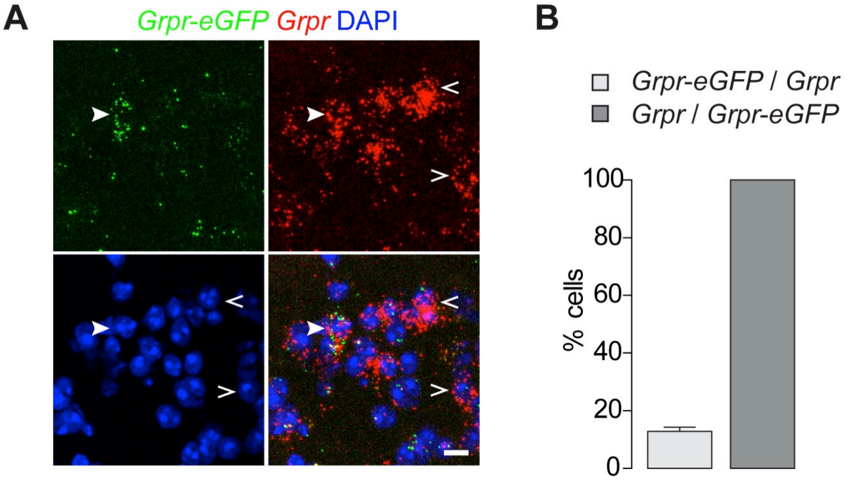

Supplemental figure 1. *Grpr* expression in *Grpr*-eGFP cells of *Grpr*::*eGFP* mice, Related to Figure 1.

(A) *In situ* hybridization on lumbar spinal cord section from *Grpr*::eGFP mouse showing that eGFP expression is restricted to *Grpr* mRNA positive neurons but only a small percentage of *Grpr* mRNA-positive neurons also express *eGFP*. Solid arrowheads indicate an eGFP positive *Grpr*-expressing neuron, open arrowheads indicate two examples of *Grpr*-expressing neurons devoid of eGFP. Scale bar, 10 µm.

(B) Quantification of double *in situ* hybridization. All analyzed *Grpr*-*eGFP* cells were positive for *Grpr* mRNA (41 out of 41 cells). Thirteen ± 1 % *Grpr* mRNA-positive cells expressed *eGFP* (41 out of 307 cells). Fifteen sections from 3 mice were analyzed (mean ± sem).

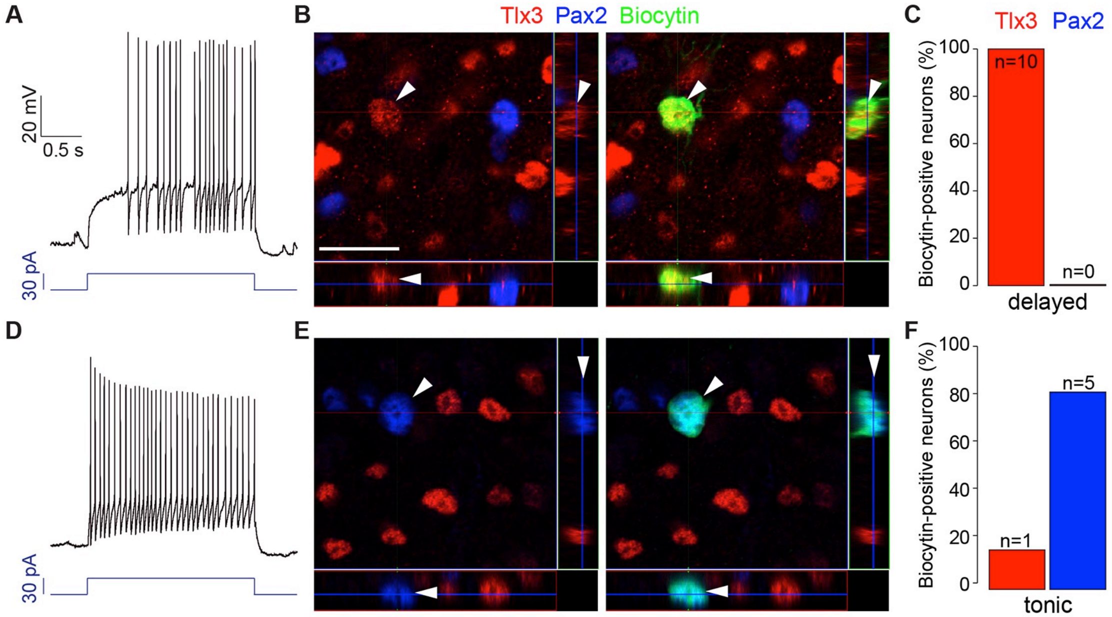

Supplemental figure2. Post-hoc neurochemical characterization of delayed and tonic firing neurons, Related to Figure1.

(A) Delayed firing pattern in a *Grpr*-eGFP neuron that was filled with biocytin during whole-cell recording.

(B) High resolution analysis of a delayed firing *Grpr*-eGFP neuron (arrowheads) that has been filled with biocytin during whole-cell recording. After completion of the recording, the slice was processed for immunostaining and reacted with streptavidin-488 (green). Co-labelling with antibodies against Tlx3 (red) and Pax2 (blue) confirms the excitatory phenotype. Scale bar, 20 μm.

(C) All the 10 cells that showed a delayed firing pattern during whole-cell recording were Tlx3-positive and Pax2-negative.

(D) Tonic firing pattern in a *Grpr*-eGFP neuron.

(E) same as (B) but tonic firing Pax2-positive neuron.

(F) same as (BC) but for tonic firing neurons.

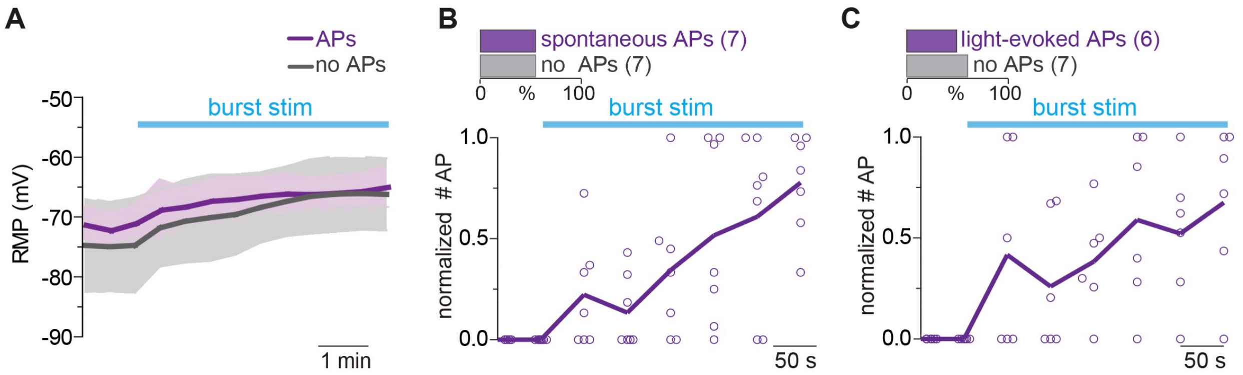

Supplemental figure 3. Time course of GRP-induced depolarization and action potential firing, Related to Figure 3.

(A) Time courses of resting membrane potential changes in *Grpr*::eGFP neurons that fired action potentials (n = 7, purple) or that did not fire action potentials (n = 7, gray) during repetitive burst stimulation of *Grp*-ChR2 neurons (blue line). Shaded lines represent mean ± sem.

(B) Top: incidence of GRPR neurons that fired spontaneous action potentials during burst-like light stimulation of GRP neurons. Bottom: scatter plot showing time course of normalized spontaneous action potentials. Open circles are individual cells (n = 7), solid line connects average values.

(C) Same as (B) but light-evoked action potentials (n = 6 cells).

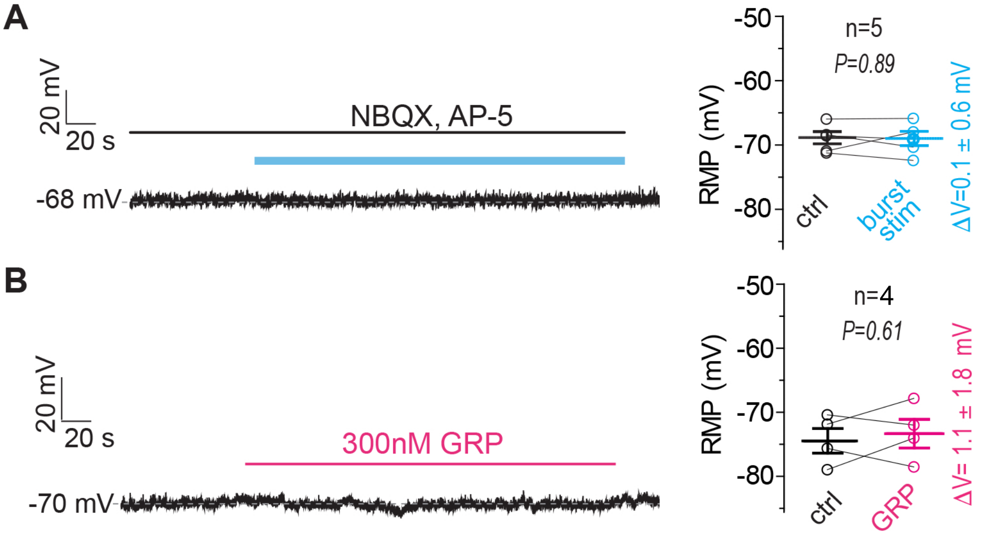

Supplemental figure 4. Tonic firing $GRPR_{inhib}$ neurons do not depolarize during repetitive burst-like stimulation of *Grp*-ChR2 neurons or superfusion with GRP, Related to Figure 3.

(A) Voltage responses in tonic firing *Grpr*-eGFP neurons elicited by repetitive burst-like stimulation of *Grp*-ChR2 neurons (blue line). Right: paired plot showing resting membrane potential (RMP) before (black) and 5 min after repetitive burst-like light stimulation (blue). Circles are individual cells (n = 5 from 5 animals; two-tailed, paired t-test, P = 0.89). Error bars indicate mean ± sem.

(B) Same as (A), but superfusion with GRP (magenta line, 300 nM) instead of presynaptic light stimulation (n = 4 from 4 mice; two-tailed, paired t-test, P = 0.61).
